# Urban–rural disparities in HPV vaccination knowledge, willingness, and uptake among women in the Tibet autonomous region: a multi-center cross-sectional study

**DOI:** 10.3389/fpubh.2026.1796545

**Published:** 2026-06-01

**Authors:** Haibei Xie, Min Zhou, Jianjun Zhang, Jingwen He, Zhilan Bai, Yan Zuo

**Affiliations:** 1 Department of Gynecology and Obstetrics Nursing, West China Second University Hospital, Sichuan University, Chengdu, China / West China School of Nursing, Sichuan University, Chengdu, China, Chengdu, China; 2 Key Laboratory of Birth Defects and Related Diseases of Women and Children (Sichuan University), Ministry of Education, Chengdu, China; 3 Outpatient Department of Tibet Autonomous Region Maternity and Children's Hospital, Lhasa, China; 4 Department of Gynecology and Obstetrics, West China Second University Hospital, Sichuan University, Chengdu, China

**Keywords:** China, HPV vaccination, knowledge, Tibetan women, uptake, urban–rural disparities, willingness, willingness-to-pay

## Abstract

**Background:**

Urban–rural disparities may hinder HPV vaccination in Tibetan regions. This study quantified disparities in HPV vaccine knowledge, willingness, and uptake among women in Tibetan settings.

**Methods:**

A multi-center cross-sectional survey of women aged 18 to 45 years was conducted. HPV knowledge was measured using a 10-item score (range 0–10). Vaccination uptake was defined as self-reported receipt of at least 1 dose. Among unvaccinated participants, willingness was dichotomized as willing versus unwilling or undecided. Multivariable linear and logistic regression models accounted for within-site correlation using cluster-robust standard errors by survey site.

**Results:**

Among 812 participants (568 urban, 244 rural), overall uptake was 29.8% (242/812) and was higher among urban than rural residents (35.2% vs. 17.2%, *p* < 0.001). The median knowledge score was 6 (IQR 2 to 7) overall; urban residents had higher knowledge than rural residents (median 6 [IQR 3 to 8] vs. 3 [IQR 0 to 6]; *p* < 0.001). Among unvaccinated participants (*n* = 570), willingness was associated with urban residence (adjusted OR 1.32, 95% CI 1.01 to 1.73), knowledge (per 2-point increase: 1.46, 1.28 to 1.67), Bachelor’s-or-higher education (1.83, 1.28 to 2.62), and prior HPV testing (1.62, 1.08 to 2.44). In the base uptake model, urban residence (1.67, 1.21 to 2.30) and knowledge (per 2-point increase: 1.34, 1.23 to 1.46) were associated with uptake. In the staged model adding prevention-service contact, Pap smear history (1.88, 1.39 to 2.56) and HPV testing history (2.41, 1.55 to 3.75) were strong predictors and the urban association attenuated (1.26, 0.93 to 1.71). Among unvaccinated participants, cost (46.7%) and safety concerns (33.7%) were common; rural residents reported substantially higher access or travel burden (47.5% vs. 7.1%) and supply or availability constraints (45.0% vs. 2.7%). The most common maximum willingness-to-pay category was 300 to 999 CNY (37.5%).

**Conclusion:**

Large urban–rural gaps in knowledge and vaccination uptake persist despite high willingness among unvaccinated women, indicating a substantial intention-to-behavior gap driven by affordability and delivery constraints. Interventions should pair education with financing and service delivery strategies that reduce travel burden and improve reliable vaccine availability in rural Tibetan settings.

## 1 Introduction

Human papillomavirus (HPV) infection is a necessary cause of virtually all cervical cancers and is also responsible for a substantial burden of other anogenital and oropharyngeal malignancies, as well as genital warts (1, 2). Cervical cancer remains a leading cause of cancer morbidity and mortality among women in many low-resource and underserved settings despite the availability of highly effective preventive tools (2, 3). Because HPV vaccination offers primary prevention and cervical screening offers secondary prevention, cervical cancer is widely recognized as one of the most preventable malignancies (2, 4). The public health challenge is increasingly defined not by the absence of effective interventions, but by inequitable delivery and uptake of prevention, especially in settings where geographic, economic, and health system constraints limit access (3, 5).

Prophylactic HPV vaccines have demonstrated strong effectiveness in preventing infection with vaccine-targeted HPV types and in reducing cervical precancerous lesions and genital warts at the population level when coverage is high (2, 6). The preventive benefits are greatest when vaccination occurs prior to sexual debut; however, catch-up vaccination among young adult women can still provide meaningful individual benefit depending on prior exposure patterns and vaccine availability (7, 8). In real-world implementation, vaccination uptake is shaped by a combination of individual knowledge and perceived risk, attitudes toward vaccine safety and effectiveness, and structural factors such as cost, service accessibility, supply stability, and convenience of appointment pathways (9, 10). In many contexts, a consistent pattern emerges in which stated willingness to vaccinate is high while actual uptake remains limited, reflecting an intention-behavior gap driven by affordability, logistics, and contact with preventive services (2, 5).

Within China, HPV vaccination has expanded over recent years, yet uptake remains uneven across provinces and population subgroups (11, 12). Differences in socioeconomic resources, health information exposure, service infrastructure, and preventive care utilization have contributed to persistent disparities between urban and rural areas (13, 14). These differences can be amplified in regions characterized by difficult terrain and long travel distances, dispersed settlements, and constrained healthcare workforces (15, 16). Tibet Autonomous Region is a particularly relevant setting in this regard (15). High-altitude geography, variable transportation access, and marked urban–rural differences in service availability create conditions in which preventive interventions may be least accessible to the women who could benefit most (11). In addition, diverse linguistic environments and local information ecosystems may influence how vaccine information is obtained and trusted, shaping knowledge, attitudes, and subsequent behavior (17).

Despite the equity relevance of Tibetan regions, evidence describing HPV-related knowledge, vaccination intent, and vaccination uptake among Tibetan women remains limited, particularly from studies designed to compare urban and rural settings within the same analytic framework (15, 18). Existing research in China has documented that HPV knowledge is frequently incomplete and that affordability and safety concerns are common reasons for hesitancy, yet fewer studies have measured knowledge, willingness, and actual behavior simultaneously in remote and minority-region contexts where structural barriers are likely to be more prominent (14, 19). Studies that do not separate intention from behavior risk mischaracterizing implementation needs, since programs designed only to improve awareness may fail if the dominant constraints are supply, service access, or affordability (15, 20). Similarly, studies that measure barriers without characterizing willingness-to-pay provide limited guidance for subsidy design or pricing strategies, especially in settings where out-of-pocket costs remain salient (11, 21).

The knowledge-attitudes-practices (KAP) framework provides a practical implementation lens for identifying modifiable bottlenecks along the pathway from awareness to intention and from intention to uptake (22, 23). Knowledge may influence perceived susceptibility and perceived benefits, attitudes may reflect safety concerns and social norms, and practices capture whether prevention is actually obtained (22, 24). Importantly, KAP measurement can illuminate whether low uptake is primarily driven by informational deficits, motivational barriers, or structural obstacles that prevent willing individuals from acting (23, 25). In HPV vaccination, this framework is particularly useful because decision-making is influenced by both personal beliefs and the local health system environment, including provider recommendation, prior contact with screening services, and the convenience of accessing vaccination points (26, 27).

In this study, we conducted a multi-center cross-sectional comparative survey among women aged 18–45 years in Tibetan settings to quantify urban–rural disparities in HPV vaccination knowledge, willingness, and uptake and to identify socio-demographic and healthcare-contact factors associated with these outcomes. The primary objective was to evaluate the current status of HPV vaccination awareness and intent and to determine factors that contribute to observed differences in vaccine uptake between urban and rural residents. Secondary objectives were to characterize item-level knowledge gaps, to describe perceived barriers among unvaccinated women, and to estimate willingness-to-pay distributions that could inform affordability-oriented intervention design. We hypothesized that urban residents would demonstrate higher knowledge and higher uptake than rural residents, that willingness would be high overall but more strongly associated with knowledge and education, and that rural residents would report higher access- and supply-related barriers. We further anticipated that indicators of preventive service contact, including prior Pap smear screening and HPV testing, would be positively associated with uptake and would attenuate the association between residence and vaccination behavior, consistent with the role of health system linkage in facilitating vaccination.

By integrating measures of knowledge, willingness, behavior, barriers, and willingness-to-pay within a single urban–rural comparative framework, this study aims to provide evidence that is directly actionable for program planning in remote Tibetan regions. A clearer understanding of where the pathway breaks down, whether at awareness, intention, or implementation logistics, can support targeted strategies such as tailored community education through trusted channels, appointment facilitation and outreach services in rural settings, integration of vaccination counseling with screening services, and pricing or subsidy approaches aligned with stated payment thresholds.

## 2 Methods

### 2.1 Study design and setting

This multi-center cross-sectional comparative study evaluated HPV vaccination KAP among women aged 18–45 years in the Tibet Autonomous Region, with a prespecified focus on urban–rural disparities. Data were collected from 15 May 2025 to 30 August 2025 across 10 survey sites representing distinct service-access contexts. Sites were selected purposively to ensure operational feasibility and to include four urban district sites, four rural township or county sites, and two peri-urban or mixed sites where access characteristics may differ from both urban cores and remote rural areas. Sites were distributed across Lhasa (urban districts) and surrounding prefecture-level areas including Shigatse, Nyingchi, Chamdo, Nagqu, and Ngari, with data collection implemented in community-facing venues that routinely serve women in the target age range, including township health centers, community service centers, and educational institutions.

### 2.2 Participants and eligibility

The target population comprised permanent female residents aged 18–45 years who had lived in their current community for at least 6 months. Eligibility required the ability to provide informed consent and to complete the questionnaire independently using a mobile device or with standardized assistance for survey navigation. Participants were excluded if they were unable to provide informed consent due to mental disorder or cognitive impairment, based on self-report and onsite screening at enrollment.

### 2.3 Residence definition

Urban versus rural residence was defined using the administrative classification of the participant’s current place of residence during the prior 6 months, consistent with the eligibility criterion. Urban residence referred to residence in an urban district or street committee area, while rural residence referred to residence in a township or village committee area. If household registration differed from current residence, classification was based on current residence to reflect local access to health services and information.

### 2.4 Sampling strategy and sample size

Quota sampling was implemented to support stable urban–rural and age-group comparisons within a geographically dispersed, site-based recruitment design. Recruitment targets were prespecified at 70% urban and 30% rural participants to ensure adequate representation of both residence strata while remaining feasible within the observed service-site attendance pattern. Age quotas were defined for five strata: 18–24 years (30%), 25–29 years (25%), 30–34 years (20%), 35–39 years (15%), and 40–45 years (10%). Enrollment continued until stratum targets were reached or the data collection period ended. Because quota sampling is non-probabilistic, prevalence estimates are interpreted as descriptive of the achieved sample, and regression estimates are interpreted as associations within the achieved sample rather than as population-causal effects.

The minimum planned sample size was calculated using a single-proportion formula adjusted for design effect:

$$n=\text{deff}\times [\frac{z_{\alpha /2}^{2}\times p\times (1-p)}{\delta^{2}}]$$

where deff (design effect) denotes the ratio of the variance under the actual sampling design to the variance under simple random sampling of the same size, accounting for the efficiency loss due to clustering within survey sites (28). For planning purposes, we assumed *p* = 0.60 for vaccination willingness, with zα/2 = 1.96, *δ* = 0.10, and deff = 1.5. A deff of 1.5 was selected as a conservative planning estimate consistent with values commonly used in multi-site health surveys where moderate within-site homogeneity is expected but cannot be estimated prior to data collection (28),which yielded a required sample size of 706. Allowing for invalid or incomplete submissions, the recruitment target was 800 participants.

### 2.5 Survey instrument development and content

A structured questionnaire was adapted from previously validated HPV knowledge-attitudes-practices (KAP) survey instruments used in Chinese populations (29–31) and revised for cultural and geographic relevance to Tibetan settings. The survey was available in Mandarin and Tibetan. Tibetan translation was performed using forward translation by bilingual staff, reconciliation by a second bilingual reviewer, and resolution of discrepancies through consensus review to ensure conceptual equivalence. The draft instrument was pilot-tested in 50 women (25 urban and 25 rural) to evaluate comprehension and response patterns. Based on pilot feedback, minor revisions were made, including simplification of technical phrasing, addition of brief clarifying descriptors for selected terms, and refinement of response labels to reduce ambiguity. Content validity was supported by the use of items derived from prior validated instruments (29–31), the bilingual translation-reconciliation process, and pilot testing for comprehension. Internal consistency of the 10-item binary knowledge scale was assessed using the Kuder–Richardson Formula 20 (KR-20), which is appropriate for dichotomous items. Split-half reliability was also assessed by correlating scores on odd-numbered versus even-numbered items, with the Spearman-Brown correction applied to estimate full-scale reliability (32).

The questionnaire captured socio-demographic characteristics including age group, ethnicity, marital status, education, occupation, monthly household income category, housing type, and medical insurance type. Information exposure was captured as the participant’s main HPV vaccine information source. Prevention-service contact included self-reported history of Pap smear screening and HPV testing, and family history of cancer.

HPV knowledge was assessed using 10 binary items scored as correct (1) or incorrect or unknown (0), yielding a total score from 0 to 10. The items were selected to capture practical domains relevant to vaccine decision-making in this setting, including transmission, prevention, timing, dose schedule, continued screening, and common misconceptions. Attitudes and practices included vaccination status and, among unvaccinated participants, willingness to receive HPV vaccination. Among unvaccinated participants, perceived barriers were assessed as a multi-select set including cost, safety concerns, access or travel burden, supply or availability constraints, and family disapproval. Willingness-to-pay was measured as an ordinal maximum amount category in RMB (<300; 300–999; 1,000–1999; 2000–3,999; ≥4,000).

### 2.6 Data collection procedures and quality assurance

Data were collected electronically through a QR-code link to a mobile questionnaire during onsite recruitment. Trained onsite staff introduced study objectives, confirmed eligibility, and explained consent procedures. Assistance was standardized to reduce interviewer effects. Staff provided technical and navigation support and, when requested due to literacy or visual constraints, read items verbatim in the participant’s preferred language without paraphrasing, interpretation, or prompting, and participants selected their own responses.

The platform enforced required fields for primary outcomes and implemented internal logic checks. *A priori* data-quality exclusions were applied. Questionnaires were excluded if any primary outcome was missing, if more than 20% of questionnaire items were missing, if completion time was less than 3 min, or if contradictory patterns were present on prespecified logic pairs. Prespecified contradiction rules included reporting HPV vaccination while simultaneously reporting never having heard of HPV vaccine information, and reporting already vaccinated while selecting an unwilling option on the willingness item intended for unvaccinated participants. When contradictions could be resolved by the platform’s skip logic, records were retained; otherwise, records were excluded.

Duplicate submissions were minimized through platform settings that restricted multiple submissions from the same device session when feasible. In addition, duplicate screening was performed by identifying records with identical site, age group, residence, and education, with a submission timestamp within 10 min; where suspected duplicates were identified, the most complete record was retained.

### 2.7 Measures and operational definitions

Education was categorized as junior high or below, high school or vocational, college or associate degree, and Bachelor’s or higher. For regression models, education was also operationalized as Bachelor’s-or-higher versus lower. Household income was categorized as <3,000, 3,000–5,999, 6,000–9,999, and ≥10,000 RMB per month and was also dichotomized as ≥6,000 versus <6,000 RMB in regression models. Insurance was categorized as urban employee insurance, resident insurance, and other or none.

HPV knowledge score was computed as the sum of 10 binary knowledge items (range 0–10). As a data integrity step, the knowledge score was recomputed from item-level responses prior to analysis and used for all analyses. For descriptive summaries, higher knowledge was defined as knowledge score ≥6.

Vaccination uptake was defined as self-reported receipt of at least one dose of any HPV vaccine. Participants were also asked whether they had received more than one dose; however, for the primary outcome, uptake was analyzed as receipt of at least one dose to reflect real-world initiation. Vaccination willingness was assessed only among unvaccinated participants. Original response options were willing, unwilling, and undecided, and willingness was dichotomized as willing versus not willing or undecided. Barrier endorsement and willingness-to-pay were analyzed among unvaccinated participants.

### 2.8 Missing data and analytic exclusions

Item-level missingness was summarized overall and by residence. After application of data-quality exclusions, regression analyses used complete-case data for variables included in each model. Multiple imputation was not performed because primary outcomes were enforced as required fields and remaining missingness was limited, and because the primary objective was to characterize patterns of association within the achieved quota-based sample.

### 2.9 Statistical analysis

Analyses were performed using R version 4.3.0. All tests were two-tailed with *α* = 0.05. Categorical variables were compared between urban and rural residents using χ^2^ tests, with Fisher’s exact tests used when expected cell counts were small. Knowledge score was summarized using medians and interquartile ranges and compared by residence using the Mann–Whitney U test. Item-level knowledge and barrier comparisons were interpreted descriptively, and *p* values for these comparisons were not adjusted for multiple testing.

Regression models addressed three outcomes: knowledge score, willingness among unvaccinated participants, and vaccination uptake in the full sample. Models accounted for within-site correlation using cluster-robust standard errors with site as the clustering unit. Because the number of clusters was modest, cluster-robust inference used a small-sample correction based on the CR2 estimator with Satterthwaite-type degrees of freedom for hypothesis tests, and conclusions were evaluated for concordance with a prespecified mixed-effects sensitivity analysis using a random intercept for site.

Knowledge score was modeled using multivariable linear regression with covariates including residence, age group, education, income, insurance, Pap smear history, HPV testing history, and family history of cancer. Among unvaccinated participants, willingness to receive HPV vaccination was modeled as a binary outcome (willing vs. unwilling or undecided) using multivariable logistic regression including residence, age group, education, income, insurance, knowledge score, Pap smear history, HPV testing history, and family history of cancer. Barrier indicators were not included as predictors of willingness because barrier endorsement may be conceptually downstream of willingness. Vaccination uptake was modeled using staged multivariable logistic regression. Model V1 included residence, age group, education, income, insurance, knowledge score, and family history of cancer. Model V2 added Pap smear history and HPV testing history to evaluate whether associations observed in Model V1 were attenuated after accounting for prevention-service contact. Odds ratios for knowledge score were presented per 2-point increase to improve interpretability. Reference categories were rural residence, age 18–24 years, education below Bachelor’s, income <6,000 RMB, and insurance other or none unless otherwise specified in tables.

### 2.10 Ethical considerations

The study protocol was ethically approved by the Ethics Committee of West China Second University Hospital, Sichuan University (approval No. 2025-511). Participants reviewed an electronic consent statement before beginning the survey, and consent was documented by an explicit confirmation step required to proceed. Participation was voluntary and could be discontinued at any time without penalty or impact on access to services. The survey was anonymous and collected no direct identifiers, and data were stored in access-controlled systems available only to authorized study personnel.

## 3 Results

### 3.1 Socio-demographic and healthcare contact characteristics

Table 1 summarizes the sociodemographic profile and prevention-service contact of 812 participants, including 568 urban residents (70.0%) and 244 rural residents (30.0%). Age distributions were similar by residence (*p* = 0.658), with most participants aged 18 to 29 years (urban 54.4% vs. rural 56.6%; total 55.0%). Marked urban–rural differences were observed across multiple characteristics. Rural participants were more often Tibetan (92.6% vs. 84.0%; *p* < 0.001) and were more likely to be married or cohabiting (64.8% vs. 46.5%), while urban participants were more commonly never married (50.7% vs. 29.5%; *p* < 0.001). Educational attainment was higher in urban settings (Bachelor’s or higher: 64.8% vs. 43.4%; *p* < 0.001), whereas rural participants more frequently reported junior high or below (13.5% vs. 3.9%) and high school or vocational education (16.8% vs. 9.0%). Occupational structure differed substantially (*p* < 0.001). Nearly half of rural participants were farmers or herders (48.0% vs. 7.0% in urban areas), while urban participants more often worked as private employees (25.9% vs. 8.2%), in healthcare (13.9% vs. 4.9%), or in government or service roles (13.4% vs. 6.6%). Rural participants also more frequently reported being homemakers or unemployed (11.9% vs. 4.9%). Consistent with this pattern, household income was lower in rural areas (*p* < 0.001): 49.2% of rural households earned <3,000 RMB per month compared with 19.4% in urban areas, while higher-income brackets were concentrated in urban settings (≥6,000 RMB: 33.9% urban vs. 4.5% rural).

**Table 1:** Participant characteristics by residence (*N* = 812).

Characteristic	Urban (*n* = 568)	Rural (*n* = 244)	Total (*N* = 812)	*p* value
Age group				0.658
18–24	175 (30.8%)	69 (28.3%)	244 (30.0%)	
25–29	134 (23.6%)	69 (28.3%)	203 (25.0%)	
30–34	113 (19.9%)	49 (20.1%)	162 (19.9%)	
35–39	90 (15.8%)	36 (14.8%)	126 (15.5%)	
40–45	56 (9.9%)	21 (8.6%)	77 (9.5%)	
Ethnicity				<0.001
Tibetan	477 (84.0%)	226 (92.6%)	703 (86.6%)	
Han	71 (12.5%)	15 (6.1%)	86 (10.6%)	
Other	20 (3.5%)	3 (1.2%)	23 (2.8%)	
Marital status				<0.001
Never married	288 (50.7%)	72 (29.5%)	360 (44.3%)	
Married/cohabiting	264 (46.5%)	158 (64.8%)	422 (52.0%)	
Divorced/separated/widowed	16 (2.8%)	14 (5.7%)	30 (3.7%)	
Education				<0.001
Junior high or below	22 (3.9%)	33 (13.5%)	55 (6.8%)	
High school/vocational	51 (9.0%)	41 (16.8%)	92 (11.3%)	
College/associate	127 (22.4%)	64 (26.2%)	191 (23.5%)	
Bachelor’s or higher	368 (64.8%)	106 (43.4%)	474 (58.4%)	
Occupation				<0.001
Student	124 (21.8%)	35 (14.3%)	159 (19.6%)	
Healthcare	79 (13.9%)	12 (4.9%)	91 (11.2%)	
Government/service	76 (13.4%)	16 (6.6%)	92 (11.3%)	
Private employee	147 (25.9%)	20 (8.2%)	167 (20.6%)	
Self-employed	74 (13.0%)	15 (6.1%)	89 (11.0%)	
Homemaker/unemployed	28 (4.9%)	29 (11.9%)	57 (7.0%)	
Farmer/herder	40 (7.0%)	117 (48.0%)	157 (19.3%)	
Monthly household income (RMB)				<0.001
<3,000	110 (19.4%)	120 (49.2%)	230 (28.3%)	
3,000–5,999	265 (46.7%)	113 (46.3%)	378 (46.6%)	
6,000–9,999	115 (20.2%)	7 (2.9%)	122 (15.0%)	
≥10,000	78 (13.7%)	4 (1.6%)	82 (10.1%)	
Housing type				<0.001
Apartment	295 (51.9%)	24 (9.8%)	319 (39.3%)	
House	164 (28.9%)	61 (25.0%)	225 (27.7%)	
Dormitory	58 (10.2%)	18 (7.4%)	76 (9.4%)	
Traditional	51 (9.0%)	141 (57.8%)	192 (23.6%)	
Medical insurance				<0.001
Urban employee	300 (52.8%)	30 (12.3%)	330 (40.6%)	
Resident	230 (40.5%)	192 (78.7%)	422 (52.0%)	
Other/none	38 (6.7%)	22 (9.0%)	60 (7.4%)	
Health history and service contact				
Family history of cancer (yes)	57 (10.0%)	20 (8.2%)	77 (9.5%)	0.409
Pap smear history (ever)	216 (38.0%)	54 (22.1%)	270 (33.3%)	<0.001
HPV testing history (ever)	102 (18.0%)	20 (8.2%)	122 (15.0%)	<0.001
Main HPV vaccine information source				<0.001
Health staff	129 (22.7%)	49 (20.1%)	178 (21.9%)	
Internet/social media	221 (38.9%)	39 (16.0%)	260 (32.0%)	
Family/friends	79 (13.9%)	29 (11.9%)	108 (13.3%)	
School	76 (13.4%)	16 (6.6%)	92 (11.3%)	
TV/radio	41 (7.2%)	41 (16.8%)	82 (10.1%)	
None	22 (3.9%)	70 (28.7%)	92 (11.3%)	

*p* values from χ^2^ tests.

Housing type and insurance coverage also reflected strong urban–rural contrasts (both *p* < 0.001). Apartments were common in urban areas (51.9%) but rare in rural areas (9.8%), where traditional housing predominated (57.8% vs. 9.0%). Urban employee insurance was reported by 52.8% of urban participants versus 12.3% of rural participants, whereas resident insurance was reported by 78.7% of rural participants versus 40.5% of urban participants. Regarding health history and service contact, family history of cancer did not differ by residence (10.0% vs. 8.2%; *p* = 0.409). In contrast, preventive service experience was less frequent among rural participants: Pap smear history (22.1% vs. 38.0%; *p* < 0.001) and HPV testing history (8.2% vs. 18.0%; *p* < 0.001). Finally, the main source of HPV vaccine information varied by residence (*p* < 0.001). Internet or social media was the dominant source among urban participants (38.9% vs. 16.0%), whereas rural participants more often cited TV or radio (16.8% vs. 7.2%) and were substantially more likely to report no information source (28.7% vs. 3.9%). Health staff were an important source in both groups (22.7% urban vs. 20.1% rural).

### 3.2 HPV vaccine knowledge

**Figure 1:**
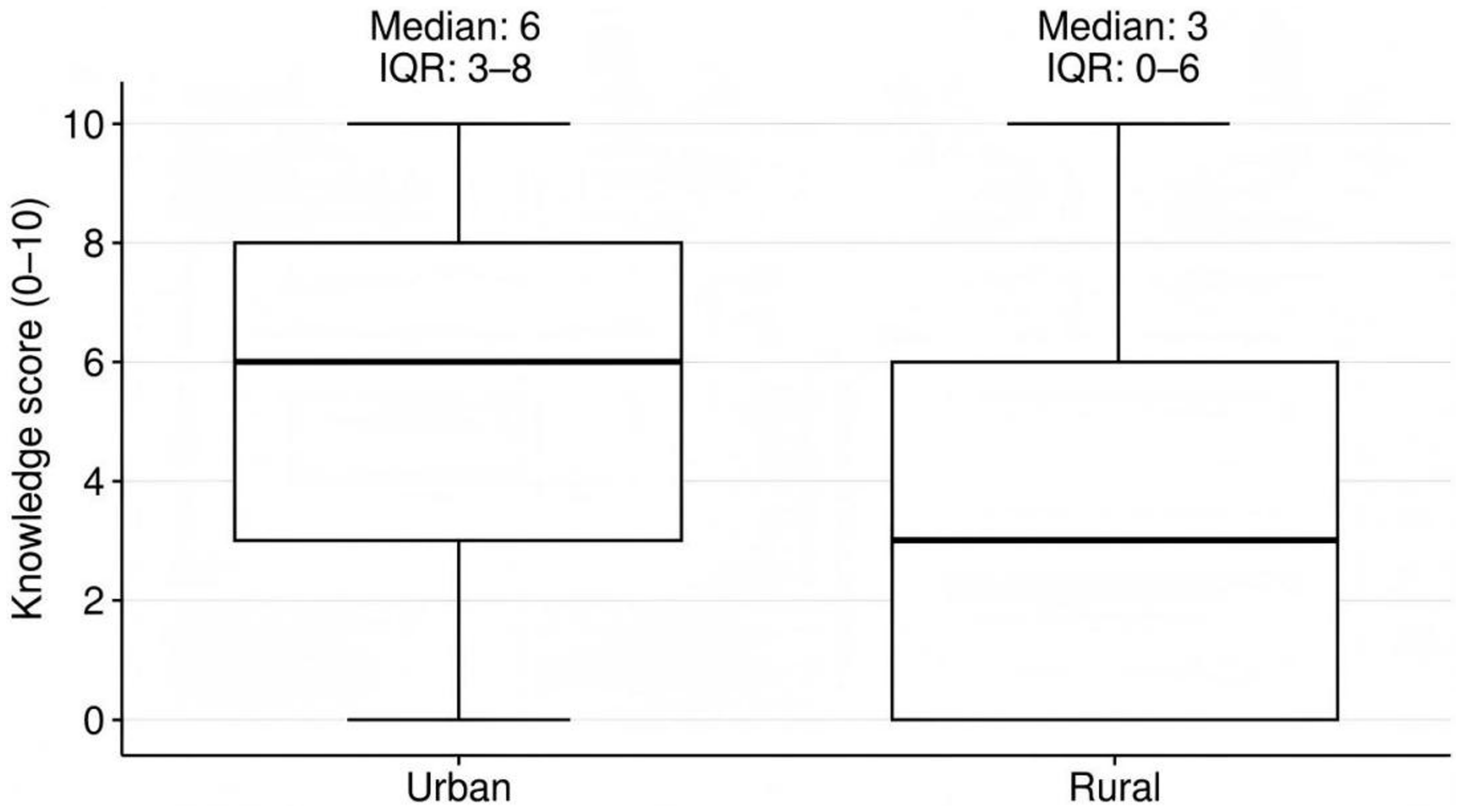
Knowledge score distribution by residence. Boxplots show the distribution of HPV knowledge scores (range 0–10) among the analytic sample (*N* = 812), stratified by residence. The overall median knowledge score was 6 (IQR 2 to 7). Urban residents had higher knowledge than rural residents (urban: median 6 [IQR 3 to 8]; rural: median 3 [IQR 0 to 6]; Mann–Whitney *U* test *p* < 0.001).

HPV vaccine knowledge showed a clear urban–rural gradient in both overall scores and item-level understanding. As shown in Figure 1, knowledge scores ranged from 0 to 10 in the analytic sample (*N* = 812). The overall median score was 6 (IQR 2 to 7), with urban residents scoring markedly higher than rural residents (urban: median 6 [IQR 3 to 8] vs. rural: median 3 [IQR 0 to 6]; Mann–Whitney U test *p* < 0.001), indicating a substantial residence-based gap in knowledge. The 10-item HPV knowledge scale demonstrated acceptable internal consistency (KR-20 = 0.72). Item-total correlations ranged from 0.24 to 0.52, indicating that all items contributed meaningfully to the composite score. The split-half reliability (Spearman-Brown corrected) was 0.69, further supporting the internal consistency of the knowledge scale.

Table 2 further characterizes specific knowledge domains underlying this difference. Across items, correct responses were consistently lower among rural participants for most topics. The largest gaps were observed in recognizing key benefits and limitations of vaccination: 58.5% of urban participants versus 36.1% of rural participants correctly identified that HPV vaccination prevents cervical cancer or genital warts (*p* < 0.001), and 50.4% versus 32.0% correctly understood that vaccination does not treat existing HPV infection (*p* < 0.001). Rural participants were also less likely to know that HPV can infect both women and men (47.9% vs. 32.8%, *p* < 0.001) and that HPV is primarily transmitted through sexual contact (83.1% vs. 59.8%, *p* < 0.001).

**Table 2:** HPV vaccine knowledge items (correct responses).

Knowledge item	Overall (*N* = 812)	Urban (*n* = 568)	Rural (*n* = 244)	*p* value
HPV vaccine prevents cervical cancer/genital warts	420 (51.7%)	332 (58.5%)	88 (36.1%)	<0.001
HPV can infect women and men	352 (43.3%)	272 (47.9%)	80 (32.8%)	<0.001
HPV is primarily transmitted through sexual contact	618 (76.1%)	472 (83.1%)	146 (59.8%)	<0.001
Most HPV infections can clear naturally	170 (20.9%)	117 (20.6%)	53 (21.7%)	0.759
Infection is not the same as disease	214 (26.4%)	172 (30.3%)	42 (17.2%)	<0.001
Adults commonly require a 3-dose regimen	558 (68.7%)	426 (75.0%)	132 (54.1%)	<0.001
Optimal timing is before first sexual encounter	458 (56.4%)	348 (61.3%)	110 (45.1%)	<0.001
Screening is still needed after vaccination	600 (73.9%)	461 (81.2%)	139 (57.0%)	<0.001
Vaccine valency refers to number of HPV types covered	208 (25.6%)	157 (27.6%)	51 (20.9%)	0.045
Vaccination does not treat existing HPV infection	364 (44.8%)	286 (50.4%)	78 (32.0%)	<0.001

Knowledge relevant to implementing vaccination and continuing prevention behaviors also differed substantially by residence. Urban participants more often correctly identified the adult 3-dose regimen requirement (75.0% vs. 54.1%, *p* < 0.001), the optimal timing of vaccination before first sexual encounter (61.3% vs. 45.1%, *p* < 0.001), and the need for continued screening after vaccination (81.2% vs. 57.0%, *p* < 0.001). Two concepts showed low correctness overall and either modest or no urban–rural difference: only 20.9% correctly noted that most HPV infections can clear naturally (20.6% urban vs. 21.7% rural, *p* = 0.759), and only 25.6% correctly understood vaccine valency (27.6% vs. 20.9%, *p* = 0.045). Finally, distinguishing infection from disease remained limited overall (26.4%), with a notable urban–rural gap (30.3% vs. 17.2%, *p* < 0.001).

### 3.3 Multivariable predictors of knowledge score

In the multivariable linear regression model for HPV knowledge score with cluster-robust standard errors by site (Table 3), urban residence remained independently associated with higher knowledge after adjustment for sociodemographic and health service factors. Compared with rural participants, urban participants had a 0.92-point higher knowledge score (*β* = 0.92, *SE* 0.16; 95% CI 0.61 to 1.23; *p* < 0.001). Education and income were also strong independent correlates of knowledge. Participants with a Bachelor’s degree or higher scored 1.55 points higher than those with lower education (*β* = 1.55, *SE* 0.18; 95% CI 1.20 to 1.90; *p* < 0.001), representing the largest effect size in the model. Higher household income (≥6,000 RMB/month) was associated with a 0.82-point higher score (*β* = 0.82, *SE* 0.17; 95% CI 0.49 to 1.15; *p* < 0.001). Insurance type showed a more modest pattern. Urban employee insurance was associated with higher knowledge compared with other or no insurance (*β* = 0.61, *SE* 0.21; 95% CI 0.20 to 1.02; *p* = 0.004), whereas resident insurance was not statistically significant (*β* = 0.31; 95% CI -0.16 to 0.78; *p* = 0.193). By contrast, age group was not significantly associated with knowledge in any category compared with ages 18 to 24 (all *p* > 0.16). Indicators of prevention-service contact showed suggestive but non-significant associations: Pap smear history was associated with a 0.18-point higher score (*p* = 0.108) and HPV testing history with a 0.29-point higher score (*p* = 0.066). Family history of cancer was not associated with knowledge (*β* = 0.12; *p* = 0.503).

**Table 3:** Multivariable linear regression for knowledge score (cluster-robust *SE* by site).

Term	*β*	*SE*	95% CI	*p* value
Residence: Urban vs. Rural	0.920	0.160	0.61 to 1.23	<0.001
Age 25–29 vs. 18–24	0.250	0.180	−0.10 to 0.60	0.163
Age 30–34 vs. 18–24	0.220	0.190	−0.15 to 0.59	0.240
Age 35–39 vs. 18–24	−0.12	0.200	−0.51 to 0.27	0.548
Age 40–45 vs. 18–24	0.180	0.220	−0.25 to 0.61	0.414
Insurance: Resident vs. Other/none	0.310	0.240	−0.16 to 0.78	0.193
Insurance: Urban employee vs. Other/none	0.610	0.210	0.20 to 1.02	0.004
Education: Bachelor’s or higher vs. lower	1.55	0.180	1.20 to 1.90	<0.001
Income: ≥6,000 RMB vs. < 6,000	0.820	0.170	0.49 to 1.15	<0.001
Pap smear history: yes vs. no	0.180	0.110	−0.04 to 0.40	0.108
HPV testing history: yes vs. no	0.290	0.160	−0.02 to 0.60	0.066
Family history of cancer: yes vs. no	0.120	0.180	−0.23 to 0.47	0.503

Reference categories: rural; age 18–24; insurance other/none; education below Bachelor’s; income <6,000 RMB.

### 3.4 Vaccination willingness among unvaccinated participants

Among unvaccinated participants (n = 570), multivariable logistic regression with cluster-robust standard errors by site showed that willingness to receive HPV vaccination was independently associated with residence, knowledge, education, and prior HPV testing (Table 4). Urban residence was associated with higher willingness compared with rural residence (adjusted OR 1.32, 95% CI 1.01 to 1.73; *p* = 0.041), indicating a modest but statistically significant urban advantage after adjustment. Knowledge was a strong predictor of willingness. For each 2-point increase in knowledge score, the odds of willingness increased by 46% (adjusted OR 1.46, 95% CI 1.28 to 1.67; *p* < 0.001). Higher education was also independently associated with willingness: participants with a Bachelor’s degree or higher had greater odds of willingness than those with lower education (adjusted OR 1.83, 95% CI 1.28 to 2.62; *p* = 0.001). Prior HPV testing was another significant correlate (adjusted OR 1.62, 95% CI 1.08 to 2.44; *p* = 0.020), suggesting that prior engagement with HPV-related services aligns with greater readiness to vaccinate. In contrast, age group was not associated with willingness (all *p* > 0.400). Insurance type and household income were also not significant predictors (resident vs. other or none: adjusted OR 1.07, *p* = 0.770; urban employee vs. other or none: adjusted OR 1.13, *p* = 0.610; income ≥6,000 RMB vs. < 6,000: adjusted OR 1.18, *p* = 0.255). Pap smear history (adjusted OR 1.09, *p* = 0.555) and family history of cancer (adjusted OR 1.30, *p* = 0.316) were not independently associated with willingness in the adjusted model.

**Table 4:** Multivariable logistic regression for willingness among unvaccinated (*n* = 570; cluster-robust *SE* by site).

Term	Adjusted OR	95% CI	*p* value
Residence: Urban vs. Rural	1.32	1.01 to 1.73	0.041
Age 25–29 vs. 18–24	1.10	0.78 to 1.55	0.586
Age 30–34 vs. 18–24	1.18	0.80 to 1.74	0.405
Age 35–39 vs. 18–24	1.14	0.72 to 1.79	0.579
Age 40–45 vs. 18–24	0.950	0.55 to 1.63	0.845
Insurance: Resident vs. Other/none	1.07	0.67 to 1.70	0.770
Insurance: Urban employee vs. Other/none	1.13	0.71 to 1.81	0.610
Knowledge score (per 2-point increase)	1.46	1.28 to 1.67	<0.001
Education: Bachelor’s or higher vs. lower	1.83	1.28 to 2.62	0.001
Income: ≥6,000 RMB vs. < 6,000	1.18	0.89 to 1.57	0.255
Pap smear history: yes vs. no	1.09	0.82 to 1.45	0.555
HPV testing history: yes vs. no	1.62	1.08 to 2.44	0.020
Family history of cancer: yes vs. no	1.30	0.78 to 2.16	0.316

Knowledge is scaled per 2-point increase to improve interpretability.

### 3.5 Vaccination uptake and predictors of vaccination behavior

**Figure 2:**
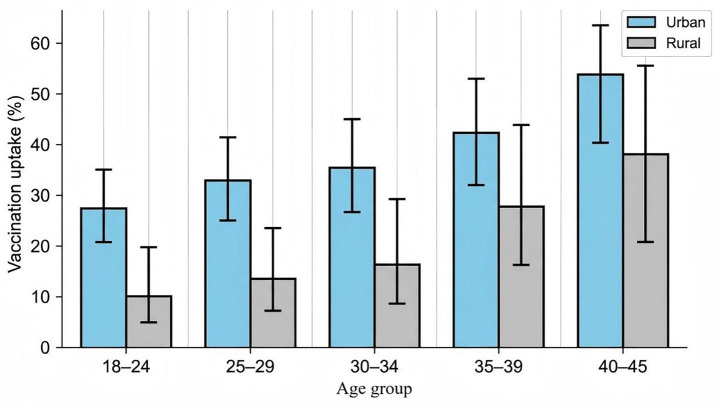
HPV vaccination uptake by age group and residence with 95% confidence intervals. Bars show HPV vaccination uptake (receipt of at least 1 dose) stratified by age group and residence among the analytic sample (*N* = 812). Uptake increased with age and was higher in urban residents than rural residents across age strata. Error bars indicate 95% confidence intervals computed using the Wilson method for binomial proportions. The overall urban–rural uptake difference in the full sample was significant (*p* < 0.001).

Figure 2 shows that HPV vaccination uptake (receipt of at least one dose) increased with age and was consistently higher among urban than rural residents across age strata, with 95% confidence intervals calculated using the Wilson method for binomial proportions. The age gradient was evident in both residence groups, indicating progressively higher uptake in older participants.

In the base multivariable model for uptake (Model V1; Table 5A) with cluster-robust standard errors by site, urban residence was associated with significantly higher odds of vaccination compared with rural residence (adjusted OR 1.67, 95% CI 1.21 to 2.30; *p* = 0.002). Uptake also increased with age, with significantly higher odds in ages 30 to 34 (adjusted OR 1.55, 95% CI 1.02 to 2.35; *p* = 0.041), 35 to 39 (2.68, 1.72 to 4.17; *p* < 0.001), and 40 to 45 (2.05, 1.20 to 3.50; *p* = 0.009) relative to ages 18 to 24, while ages 25 to 29 did not differ significantly (*p* = 0.23). Higher knowledge remained a strong independent predictor (per 2-point increase: adjusted OR 1.34, 95% CI 1.23 to 1.46; *p* < 0.001), and higher income (≥6,000 RMB/month) was also associated with uptake (adjusted OR 1.52, 95% CI 1.12 to 2.07; *p* = 0.007). Insurance type, education level, and family history of cancer were not significant in Model V1.

**Table 5A:** Multivariable logistic regression for vaccination uptake (Model V1 base; *N* = 812; cluster-robust SE by site).

Term	Adjusted OR	95% CI	*p* value
Residence: Urban vs. Rural	1.67	1.21 to 2.30	0.002
Age 25–29 vs. 18–24	1.22	0.88 to 1.70	0.230
Age 30–34 vs. 18–24	1.55	1.02 to 2.35	0.041
Age 35–39 vs. 18–24	2.68	1.72 to 4.17	<0.001
Age 40–45 vs. 18–24	2.05	1.20 to 3.50	0.009
Insurance: Resident vs. Other/none	1.10	0.69 to 1.75	0.680
Insurance: Urban employee vs. Other/none	1.06	0.68 to 1.65	0.794
Knowledge score (per 2-point increase)	1.34	1.23 to 1.46	<0.001
Education: Bachelor’s or higher vs. lower	1.15	0.82 to 1.62	0.417
Income: ≥6,000 RMB vs. < 6,000	1.52	1.12 to 2.07	0.007
Family history of cancer: yes vs. no	1.28	0.86 to 1.90	0.226

After adding prevention-service contact variables (Model V2; Table 5B), the association between urban residence and uptake attenuated and was no longer statistically significant (adjusted OR 1.26, 95% CI 0.93 to 1.71; *p* = 0.135). The age gradient persisted, with higher odds of uptake in ages 30 to 34 (1.63, 1.06 to 2.51; *p* = 0.026), 35 to 39 (2.49, 1.55 to 4.01; *p* < 0.001), and 40 to 45 (1.91, 1.11 to 3.30; *p* = 0.019) compared with ages 18 to 24. Knowledge remained independently associated, though with a slightly smaller effect size (per 2-point increase: adjusted OR 1.26, 95% CI 1.16 to 1.37; *p* < 0.001). Higher income remained significant (adjusted OR 1.39, 95% CI 1.02 to 1.90; *p* = 0.038). Prior prevention-service contact was also associated with uptake: Pap smear history (95% CI 1.39 to 2.56; *p* < 0.001) and HPV testing history (95% CI 1.55 to 3.75; *p* < 0.001). Insurance type, education, and family history of cancer remained non-significant in the fully adjusted model. This staged analysis is interpreted cautiously as evidence of health-service linkage rather than formal mediation.

**Table 5B:** Multivariable logistic regression for vaccination uptake (Model V2 adds prevention-service contact; *N* = 812; cluster-robust *SE* by site).

Term	Adjusted OR	95% CI	*p* value
Residence: Urban vs. Rural	1.26	0.93 to 1.71	0.135
Age 25–29 vs. 18–24	1.29	0.93 to 1.80	0.126
Age 30–34 vs. 18–24	1.63	1.06 to 2.51	0.026
Age 35–39 vs. 18–24	2.49	1.55 to 4.01	<0.001
Age 40–45 vs. 18–24	1.91	1.11 to 3.30	0.019
Insurance: Resident vs. Other/none	1.08	0.67 to 1.74	0.755
Insurance: Urban employee vs. Other/none	1.02	0.65 to 1.62	0.930
Knowledge score (per 2-point increase)	1.26	1.16 to 1.37	<0.001
Education: Bachelor’s or higher vs. lower	1.07	0.76 to 1.50	0.696
Income: ≥6,000 RMB vs. < 6,000	1.39	1.02 to 1.90	0.038
Family history of cancer: yes vs. no	1.21	0.81 to 1.81	0.353
Pap smear history: yes vs. no	1.88	1.39 to 2.56	<0.001
HPV testing history: yes vs. no	2.41	1.55 to 3.75	<0.001

Model V2 is presented as a staged analysis incorporating prevention-service contact and is interpreted as health-system linkage rather than causal mediation.

### 3.6 Barriers to vaccination among unvaccinated participants

**Figure 3:**
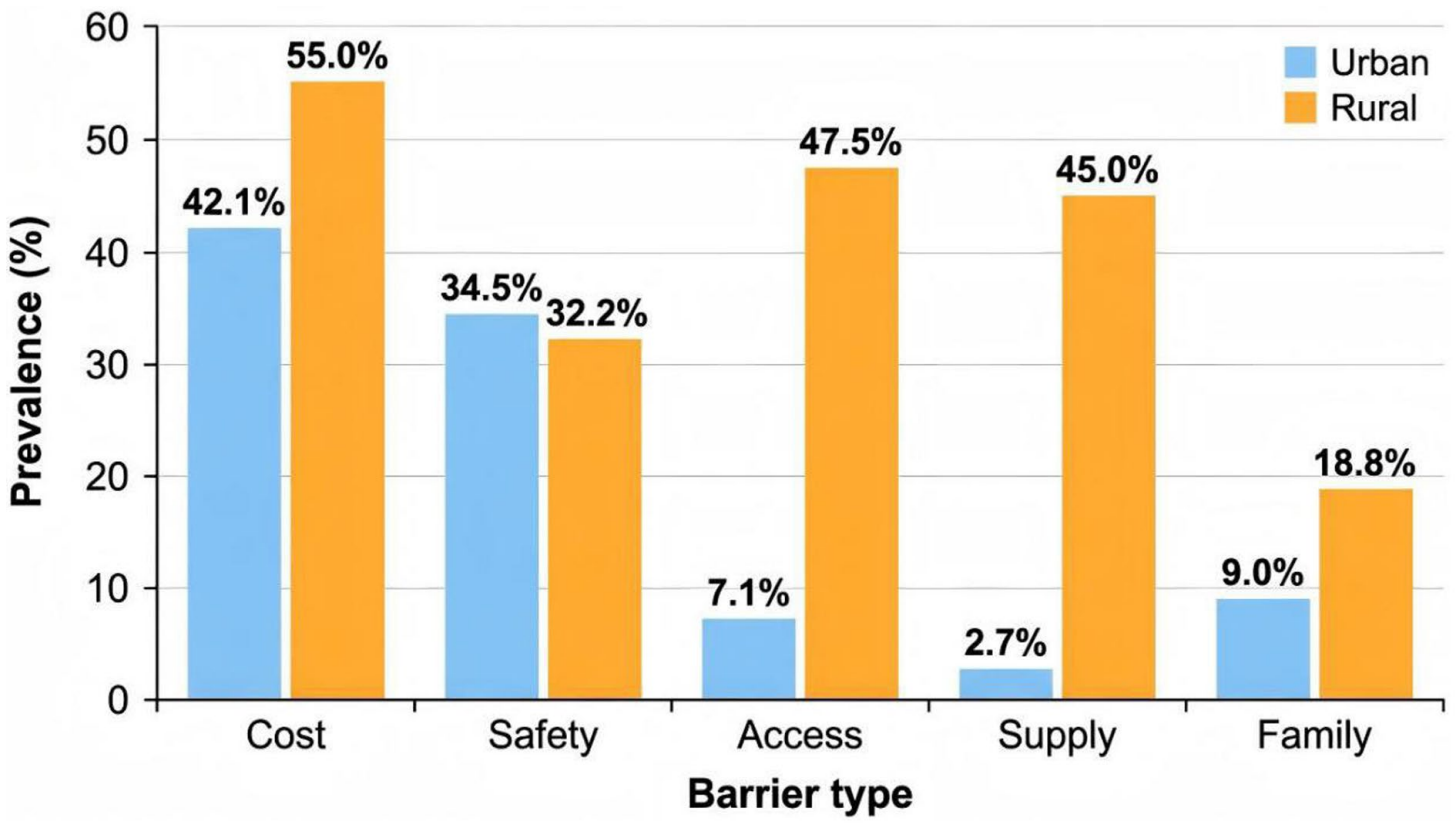
Vaccination barriers among unvaccinated participants by residence. Among unvaccinated participants (*N* = 570), the prevalence of self-reported barriers to HPV vaccination is shown by residence. Barriers were assessed as multi-select responses. Cost and safety concerns were common in both strata. Rural residents reported substantially higher access or travel burden and supply or availability constraints compared with urban residents, and family disapproval was also more frequent in rural residents. Percentages are calculated using the unvaccinated denominator within each residence group (urban *n* = 368; rural *n* = 202).

Figure 3 and Table 6 summarize self-reported barriers to HPV vaccination among unvaccinated participants (*N* = 570), using residence-specific denominators (urban *n* = 368; rural *n* = 202) and allowing multiple selections. Overall, cost was the most frequently reported barrier (46.7%), followed by safety concerns (33.7%). Clear urban–rural differences emerged for several structural and social barriers.

**Table 6:** Barriers to vaccination among unvaccinated (multi-select; *N* = 570).

Barrier	Urban (*n* = 368)	Rural (*n* = 202)	Total (*N* = 570)	*p* value
Cost	155 (42.1%)	111 (55.0%)	266 (46.7%)	0.003
Safety concerns	127 (34.5%)	65 (32.2%)	192 (33.7%)	0.568
Access/travel burden	26 (7.1%)	96 (47.5%)	122 (21.4%)	<0.001
Supply/availability	10 (2.7%)	91 (45.0%)	101 (17.7%)	<0.001
Family disapproval	33 (9.0%)	38 (18.8%)	71 (12.5%)	<0.001

Multi-select; percentages use unvaccinated denominator per stratum.

Cost concerns were common in both strata but were significantly more prevalent among rural residents than urban residents (55.0% vs. 42.1%; *p* = 0.003). Safety concerns were also frequently reported, but did not differ by residence (32.2% rural vs. 34.5% urban; *p* = 0.568), suggesting broadly shared apprehension about vaccine safety. In contrast, access-related barriers were strongly concentrated in rural residents. Nearly half of rural participants reported access or travel burden (47.5%) compared with 7.1% of urban participants (*p* < 0.001). Similarly, vaccine supply or availability constraints were reported by 45.0% of rural residents but only 2.7% of urban residents (*p* < 0.001). These large differences indicate that rural participants face substantial logistical and delivery constraints beyond individual-level concerns. Family disapproval was less common than cost or safety concerns overall (12.5%) but was significantly more frequent among rural residents than urban residents (18.8% vs. 9.0%; *p* < 0.001), highlighting a potentially more restrictive social context in rural settings. Taken together, the barrier profile suggests that while affordability and safety perceptions are widespread, rural–urban disparities are largely driven by structural access, supply limitations, and, to a lesser extent, family-level opposition.

### 3.7 Willingness-to-pay among unvaccinated participants

**Figure 4:**
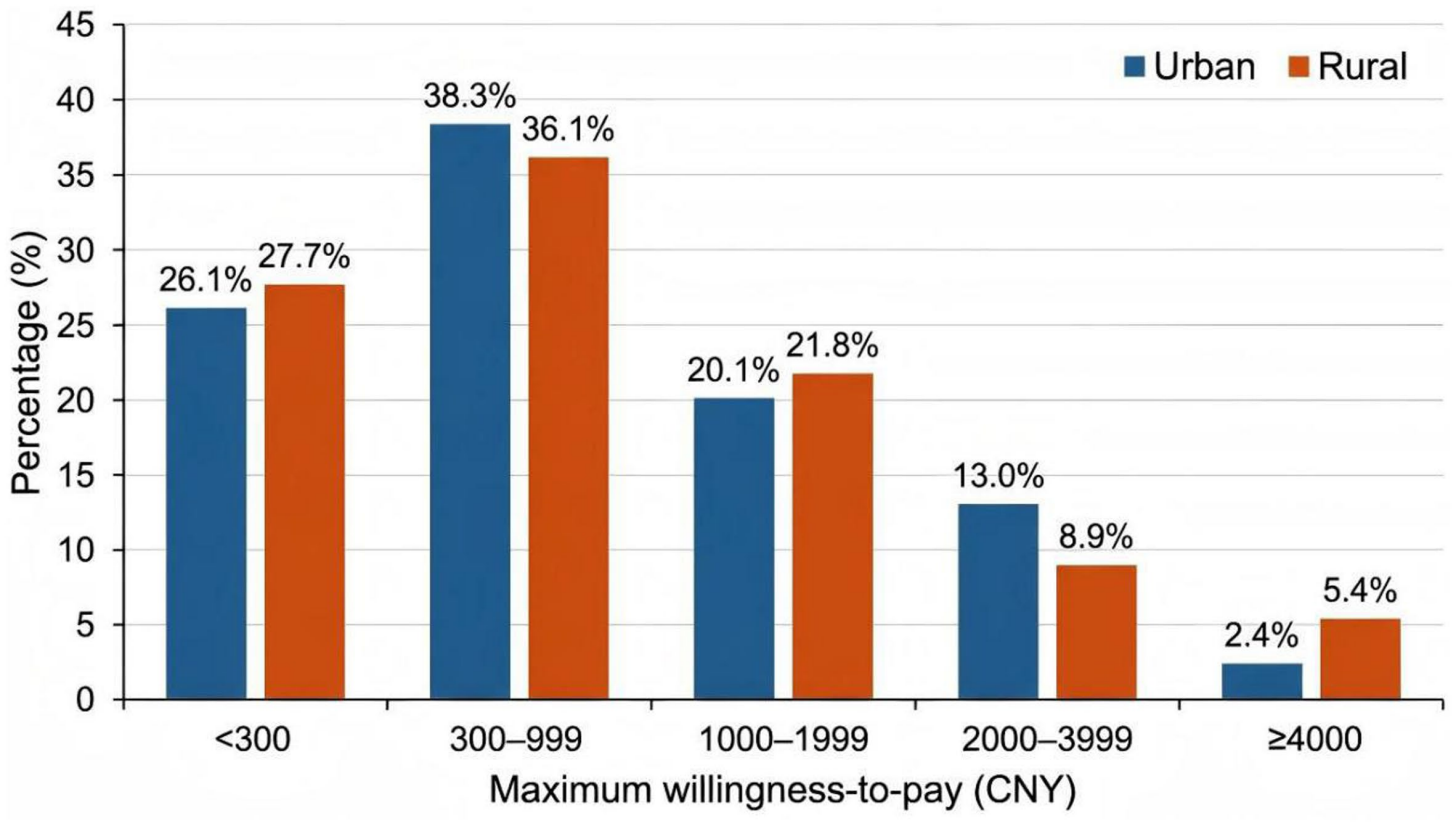
Willingness-to-pay distribution among unvaccinated participants by residence. Among unvaccinated participants (*N* = 570; urban *n* = 368, rural *n* = 202), maximum willingness-to-pay for HPV vaccination is shown by residence. The most common category was 300–999 CNY in both strata. Overall differences in willingness-to-pay distributions between urban and rural residents were modest. Percentages are calculated using the unvaccinated denominator within each residence group.

Figure 4 and Table 7 describe maximum willingness-to-pay (WTP) for HPV vaccination among unvaccinated participants (*N* = 570), calculated within residence-specific denominators (urban n = 368; rural *n* = 202). Across the full unvaccinated sample, the most common maximum WTP category was 300 to 999 CNY (37.5%), followed by <300 CNY (26.7%) and 1,000 to 1999 CNY (20.7%). Higher WTP categories were less frequent overall, with 11.6% reporting 2000 to 3,999 CNY and 3.5% reporting ≥4,000 CNY.

**Table 7:** Maximum willingness-to-pay among unvaccinated (*N* = 570).

Maximum willingness-to-pay (CNY)	Urban (*n* = 368)	Rural (*n* = 202)	Total (*N* = 570)
<300	96 (26.1%)	56 (27.7%)	152 (26.7%)
300–999	141 (38.3%)	73 (36.1%)	214 (37.5%)
1,000–1999	74 (20.1%)	44 (21.8%)	118 (20.7%)
2000–3,999	48 (13.0%)	18 (8.9%)	66 (11.6%)
≥4,000	9 (2.4%)	11 (5.4%)	20 (3.5%)

The distributions were broadly similar between urban and rural residents, indicating only modest residence differences in stated affordability thresholds. In both strata, 300 to 999 CNY was the modal category (38.3% urban vs. 36.1% rural). The proportion willing to pay <300 CNY was also comparable (26.1% urban vs. 27.7% rural), as was the proportion willing to pay 1,000 to 1999 CNY (20.1% vs. 21.8%). Small differences appeared in the upper ranges: urban participants more often selected 2000 to 3,999 CNY (13.0% vs. 8.9%), whereas rural participants slightly more often selected ≥4,000 CNY (5.4% vs. 2.4%). Overall, the pattern suggests a broadly similar willingness-to-pay profile across residence groups, with most unvaccinated participants indicating a maximum WTP below 2000 CNY.

Overall, urban residents demonstrated higher HPV vaccine knowledge and higher HPV vaccination uptake than rural residents. Knowledge level was strongly associated with both vaccination willingness and uptake, while rural residents reported substantially higher access and supply barriers among unvaccinated participants. Household income and age were also associated with uptake, supporting the combined influence of socio-economic capacity and life-stage factors on vaccine behavior.

## 4 Discussion

This multi-center cross-sectional comparative study evaluated HPV vaccination knowledge, attitudes, and practices among women aged 18 to 45 years in Tibetan settings, with a prespecified focus on urban–rural disparities. A consistent pattern emerged across the pathway from awareness to action. Urban participants demonstrated higher HPV vaccine knowledge and higher vaccination uptake, while willingness to vaccinate among unvaccinated women remained high in both residence strata. The gap between willingness and uptake indicates a substantial intention to behavior gap, and the combination of high intent with low uptake points to implementation constraints that extend beyond motivation.

The knowledge gradient by residence is consistent with a broad evidence base from China and other settings showing that HPV-related knowledge is patterned by education, socioeconomic resources, and information access (33, 34). In this study, higher educational attainment and higher household income were associated with higher knowledge scores, and urban residents were more likely to report school-based or internet-based information sources (35, 36). Rural residents more often reported no identifiable information source or reliance on broadcast channels (34). This information environment likely contributes to both lower baseline knowledge and slower correction of misconceptions (37). From an implementation standpoint, the item-level profile matters as much as the total score (34). Low correct response rates on items involving natural clearance, vaccine scope, and the need for screening after vaccination represent gaps that can directly impair risk appraisal and can foster either false reassurance or unnecessary fear (34, 38). The practical implication is that education should emphasize clear, behavior-linked concepts rather than abstract facts, particularly the preventive nature of vaccination, the persistence of screening needs, and realistic framing of HPV infection as common yet usually transient (33, 39).

Willingness among unvaccinated women was high overall and strongly associated with knowledge and education (40, 41). This pattern matches KAP research where intention rises as understanding of HPV-related disease burden and vaccine benefit increases and as uncertainty declines (23, 40). The independent association between education and willingness after adjusting for knowledge suggests that education captures determinants beyond factual knowledge, including trust in biomedical prevention, the ability to evaluate competing information, and navigation capacity for appointments and payment. This also implies that even if knowledge campaigns improve scores, structural barriers will still limit translation into behavior among women who lack time, money, or access pathways (33, 36). The modest positive association between preventive service contact and willingness supports the idea that clinical touchpoints can strengthen intention through provider recommendation and perceived legitimacy of prevention (42, 43).

The core policy signal is the intention to behavior gap paired with a marked rural disadvantage in uptake (15, 44). Uptake remained far below willingness, and the gap was larger in rural settings (13, 45). The barrier profile among unvaccinated women supports a structural interpretation (15, 20). Cost and safety concerns were common in both strata, while rural participants reported substantially higher travel burden, access difficulty, and supply constraints, along with greater family disapproval (15, 46). A pattern of high willingness plus prominent access and supply barriers is typical of settings where the dominant constraints are delivery and affordability rather than demand (20, 47). In practice, this indicates that purely informational interventions, even if successful at improving knowledge, are unlikely to close the rural uptake gap unless paired with delivery changes that reduce travel, waiting time, and uncertainty about availability (17, 48).

Affordability appears to be a shared constraint across strata. Cost was the most frequently endorsed barrier, and willingness-to-pay clustered in low to mid price thresholds, with only a small minority indicating the highest categories (49, 50). This distribution implies that market pricing for multi-dose series will exclude a large share of potential vaccine recipients without subsidy or reimbursement, particularly when indirect costs such as transport and lost work time are considered (15, 51). The modest residence difference in willingness-to-pay suggests that affordability constraints are widespread rather than confined to rural households, although household income gradients still matter. For program design, willingness-to-pay results can inform pragmatic subsidy thresholds and can support tiered financing approaches (21, 52). A policy that reduces the effective price at point of care, combined with predictable availability, is more likely to convert willingness into uptake than a policy that focuses only on awareness (49, 53).

The staged uptake modeling provides additional insight into plausible pathways producing urban–rural disparities. In the base model, residence, age, income, and knowledge were associated with uptake, consistent with a combined pathway in which resources and information facilitate preventive action (13, 45). After adding Pap smear history and HPV testing history, the residence association attenuated and preventive service contact variables were strongly associated with uptake (15, 20). This pattern is plausible for several reasons. Preventive service contact can reflect better geographic access, more frequent interactions with clinicians, and higher baseline health-seeking behavior (54, 55). It can also create opportunities for recommendation and assisted scheduling, which are known to be strong proximal drivers of vaccine behavior (43, 56). The attenuation observed after adjustment for service contact is consistent with the interpretation that part of the urban advantage operates through higher linkage to prevention services (57). This supports a program logic in which screening and vaccination are integrated, not only conceptually but operationally, so that clinic contact is used as a platform for counseling, eligibility clarification, and facilitated referral (54, 57).

The age gradient in uptake also merits interpretation. Uptake increased with age even after adjustment. Adult age patterns for HPV vaccination vary across studies, reflecting differences in policy timing, cohort differences in education and information environments, and variation in reproductive health service utilization (58, 59). In Tibetan settings, higher uptake among older women may plausibly reflect cumulative clinic contact, including antenatal care, postpartum services, contraception counseling, and screening visits (54). Such contact increases the opportunity for provider recommendation and can reduce logistical barriers through guided referral pathways (43). An age gradient can also reflect differential affordability and stability, since older women may have more consistent income or insurance coverage (45). For program planning, the age pattern suggests that integration with reproductive health and screening services may be particularly effective, while younger women may require alternative access routes such as school or workplace outreach plus simplified appointment and subsidy mechanisms (17).

Safety concerns were prevalent in both strata and represent a modifiable barrier. Vaccine confidence is shaped by trust, perceived transparency, and messenger credibility (60, 61). Generic reassurance is rarely sufficient in settings where misinformation circulates or where adverse event stories are salient (62, 63). Communication with stronger evidence includes structured counseling that explains common mild reactions, frames rare serious adverse events accurately, clarifies contraindications, and explicitly addresses misunderstandings about fertility and sexual behavior (64, 65). Because health staff are an important information source, strengthening provider communication skills and ensuring consistent messaging can yield high leverage (64, 66). In rural settings, where provider contact may be less frequent, trained community health workers and township clinicians can play a key role if they are equipped with standardized scripts and referral pathways (67, 68).

The information environment findings complement the knowledge and barrier results and help explain persistence of residence gradients. Urban participants cited internet or school sources more often, which increases reach but can also expose individuals to conflicting narratives (17, 69). Rural participants more often reported no information source and greater reliance on broadcast channels, suggesting an information access gap in addition to service access gaps (15, 70). Intervention design should differ by context. Urban settings may benefit from misinformation-resistant digital communication and school-based education that emphasizes safety, schedule, and continued screening (19, 48). Rural settings may benefit from community-based outreach delivered through trusted local messengers in Tibetan language, with messaging that is tightly linked to practical steps for obtaining vaccination and with clear information on cost and availability (65, 67). Improving information quality without improving access risks increasing frustration among willing women who cannot obtain vaccination, so coordination between communication and delivery is essential (15, 20).

Taken together, the results indicate that reducing the urban–rural uptake gap will require coordinated action on affordability, service access, supply stability, and information quality. Knowledge improvement is important for strengthening willingness and for correcting misconceptions, but rural constraints related to travel burden and vaccine availability appear decisive for behavior. Delivery models that reduce indirect costs are likely to be high yield, including scheduled outreach vaccination sessions, mobile vaccination services, and reliable stock management at township level. Financial strategies, including targeted subsidies or reimbursement mechanisms, are likely to be necessary to shift effective prices into ranges that align with stated willingness-to-pay thresholds. Integration with screening services offers a pragmatic route for both counseling and facilitated referral and may partially offset structural constraints by leveraging existing clinic contact.

### 4.1 Limitations and implications

Quota sampling and purposive site selection limit representativeness, so prevalence estimates should be interpreted as descriptive of the achieved sample rather than population estimates for the entire region. Vaccination status and prevention-service history were self-reported and may introduce misclassification. The cross-sectional design limits causal interpretation and cannot establish temporal ordering among knowledge, clinical contact, and vaccination behavior. Residual confounding is possible, including factors not measured in the survey such as parity, partner influence, and provider recommendation strength. Finally, although the knowledge scale demonstrated acceptable internal consistency (KR-20 = 0.72; Spearman-Brown split-half = 0.69) and content validity was supported through derivation from prior instruments, bilingual translation, and pilot testing, a formal construct validity analysis (e.g., exploratory factor analysis) was not conducted.

The findings support interventions that pair education with structural access improvements. Rural uptake shortfalls align with travel burden and supply constraints, so outreach delivery, mobile services, and stable vaccine availability at township level are likely to yield larger gains than education alone. Cost remains a primary barrier across residence strata, and willingness-to-pay profiles can inform subsidy levels that move effective prices into ranges more women consider feasible. Prevention-service contact represents a practical platform for counseling and facilitated referral that can reduce the intention to behavior gap.

## 5 Conclusion

HPV vaccination coverage among women aged 18 to 45 years in Tibet remains constrained by both informational and structural barriers. Urban residents demonstrate higher HPV vaccine knowledge and higher vaccination uptake, while willingness among unvaccinated women is high in both urban and rural strata, indicating a substantial intention to behavior gap. Knowledge and education are closely linked to willingness, and vaccination uptake is associated with knowledge, age, and household income, with rural women facing additional constraints related to access, travel burden, and supply availability. The attenuation of the residence association after accounting for prevention-service contact suggests that linkage to screening and HPV testing services is an important pathway facilitating uptake. Program strategies most likely to reduce disparities should combine context-adapted education that addresses key misconceptions and safety concerns with delivery reforms that reduce travel and scheduling burden, strengthen supply stability in rural areas, and lower out-of-pocket costs through subsidies or reimbursement mechanisms. Integrating vaccination counseling and referral into cervical screening and reproductive health services offers a pragmatic approach to convert high willingness into actual uptake and to narrow urban–rural inequities in HPV prevention.

## Data Availability

The raw data supporting the conclusions of this article will be made available by the authors, without undue reservation.
